# Thyroid V40 is a good predictor for subclinical hypothyroidism in patients with nasopharyngeal carcinoma after intensity modulated radiation therapy: a randomized clinical trial

**DOI:** 10.1186/s13014-023-02329-x

**Published:** 2023-08-25

**Authors:** Yun Xu, Hewei Peng, Guangjian Su, Yanming Cheng, Qiaojuan Guo, Lanyan Guo, Xian-E Peng, Jiangfeng Ke

**Affiliations:** 1 Department of Radiation Oncology, Clinical Oncology School of Fujian Medical University, Fujian Cancer Hospital, Fuzhou, China; 2 Department of Epidemiology and Health Statistics, Fujian Provincial Key Laboratory of Environment Factors and Cancer, School of Public Health, Fujian Medical University, Fuzhou, China; 3 Department of Clinical Laboratory, Clinical Oncology School of Fujian Medical University, Fujian Cancer Hospital, Fuzhou, China; 4 Key Laboratory of Ministry of Education for Gastrointestinal Cancer, Fujian Medical University, Fuzhou, China; 5 School of Medical Imaging, Fujian Medical University, Fuzhou, China

**Keywords:** Subclinical hypothyroidism, IMRT, Nasopharyngeal carcinoma

## Abstract

**Background:**

Hypothyroidism (HT) and subclinical HT after radiotherapy is frequent in nasopharyngeal carcinoma (NPC) patients, results in negative impact on patients' quality of life. The percentage of thyroid volume receiving more than 40 Gy (V40) ≤ 85% was reported to be a useful dose constraint to adopt during intensity-modulated radiation therapy (IMRT) planning. This study aims to verify whether V40 ≤ 85% can be used as an effective dose constraint in IMRT planning in a randomized clinical trial.

**Methods:**

This single-center 1:1 randomized clinical trial was conducted in Fujian province hospital between March 2018 and September 2022. All patients were treated with IMRT and randomized to induction chemo followed by concurrent chemo-IMRT or concurrent chemo-IMRT alone. Ninety-two clinically NPC patients were included in this study. The thyroid function tests were performed for all patients before and after radiation at regular intervals. Thyroid dose-constraint was defined as V40 ≤ 85%. The primary outcome in this study was subclinical HT.

**Results:**

Median follow up was 34 months. Significant difference in the incidence of subclinical HT between the thyroid dose-constraint group and unrestricted group was observed (*P* = 0.023). The risk of subclinical HT in the thyroid dose-constraint group was lower than that in the unrestricted group (*P* = 0.022). Univariate and multivariate cox regression analysis indicated that thyroid dose-constraint was a protective effect of subclinical HT (HR = 0.408, 95% CI 0.184–0.904; HR_adjusted_ = 0.361, 95% CI 0.155–0.841).

**Conclusion:**

V40 ≤ 85% can be used as an effective dose constraint in IMRT planning to prevent radiation-induced subclinical HT.

## Introduction

Nasopharyngeal carcinoma (NPC) is prevalent in Southern China. Intensity-modulated radiation therapy (IMRT) is the primary treatment, with a 5-year overall survival (OS) rate more than 80% [1–3]. With the improvement of survival rate and the prolongation of survival time, more attention has been paid to the quality of life of NPC patients. Although IMRT is superior to conventional radiotherapy in terms of target dose coverage and normal tissue protection, it increases the incidence of hypothyroidism (HT) and shortens the latency period [4, 5]. HT after radiotherapy of head and neck is frequent, occurring in 20 to 79% of patients [6, 7]. Subclinical HT is manifested by fatigue, cold tolerance, dry skin, and weight gain, as well as mild impairments in declarative, working memory, and mood [8, 9]. Subclinical HT is associated with an increased risk of heart disease, which has a significant negative impact on the quality of life of patients [8]. Fan et al. [10] recommend that regular clinical and serum thyroid function tests are essential among NPC survivors after radiotherapy. The risk of hypothyroidism after radiotherapy was significantly higher in NPC patients than in the general population of Taiwan and head and neck cancer patients. However, thyroid hormone testing is not a routine test for NPC patients.

Several studies have focused on radiation-induced HT. Bhandare et al. indicated that the total dose to the thyroid was significantly associated with clinical primary HT [11]. Zhai et al. found that the mean dose constraint for the thyroid was about 45 Gy and suggested that the optimization goal of the plan was to reduce the thyroid The percentage of thyroid volume receiving more than 45 Gy (V45) to 0.5 and V50 to 0.35 [4]. Huang et al. recommended that V25 ≤ 60%, V35 ≤ 55%, V45 ≤ 45% as the "strict" dose-volume histogram lines, and V25 > 95%, V35 > 90%, V45 > 75% as the "inhibition" dose-volume histogram lines without damaging the target coverage [12]. Nevertheless, there is no clear guidance on dose-limiting protection of the thyroid gland in the RTOG guidelines. It is urgent to investigated the optimal dose-volume thresholds of the thyroid gland to prevent the incidence of HT and guide individualized treatment.

Sommat et al. [13] presented that V40 ≤ 85% can be a useful dose constraint to adopt during IMRT planning without compromising tumor coverage. The area under receiver operating characteristics curve was 0.69. And the incidence of radiation-induced HT in the group with V40 ≤ 85% and V40 > 85% was 21.4% and 61.4% respectively. Therefore, this study aims to verify whether V40 ≤ 85% can be used as an effective dose constraint in IMRT planning in a randomized clinical trial.

## Methods

### Participants

This single-center study was conducted in Fujian cancer hospital, Fuzhou, China. Patients with newly pathologically confirmed NPC, age 18 to 70 years, with Eastern Cooperative Oncology Group performance status (ECOG PS) between 0 and 1, who provided written consent to participate in the study, cooperated with regular follow-up and complied with the experimental requirements were included in this study. Exclusion criteria were as follows: (1) patients with severe cardiovascular disease or other underlying conditions that affect the implementation of standardized treatment for NPC; (2) patients with previous diseases of thyroid, hypothalamus and pituitary; (3) patients have received prior chemotherapy or radiotherapy of the head and neck; (4) patients with other malignancies. All participants signed a written informed consent form.

### Trial design and procedure

The trial was conducted between March 2018 and September 2022. Participants were recruited between March 2018 and December 2019. A total of 92 patients met our criteria were enrolled and assigned to the thyroid dose-volume constraint and non-restricted groups (1:1) using simple randomization. (1) The serial numbers of 92 subjects were proposed; (2) Numbers were randomly generated; (3) Subjects with odd random numbers were assigned to group A and even numbers to group B; (4) The thyroid dose volume was restricted in group A and not in group B.

Patients with stage T1N0M0/T2N0M0 received radical radiotherapy alone. Patients with T1N1M0/T2N1M0 were treated with single agent chemotherapy combined with radiotherapy. Stage III-IVB patients were treated with a combination regimen that included concurrent and/or sequential radiotherapy and cisplatin-based chemotherapy. Radiotherapy was performed using 6 MV photons via IMRT. The thyroid dose-volume constraint group was defined as the percentage of thyroid volume receiving more than 40 Gy (V40) less than 85%. The non-restricted group was not restricted. Furthermore, CT and MRICT fusion planning was performed for all patients with co-registration software (Oncentra MasterPlan version 1.5, Nucletron B.V., Veenendaal, The Netherlands).

Sample size was calculated based on historical data suggesting an incidence of 21.4% and 50.4% in the dose-constraint and unrestricted group, respectively. Assuming a dropout rate of 10%, a sample size of 90 patients (45 in each group) was required with 80% power and two-sided α of 0.05.

### Thyroid function evaluation

The thyroid function including thyroid stimulating hormone (TSH), free thyroxine (FT4), and free triiodothyronine (FT3) measurements were performed for all patients before radiation, at the end of radiotherapy and at an interval of 3–6 months in the first two years. Thereafter, they were followed up every 6 months from the third to the fifth year and annually afterwards. Serum TSH, FT4, and FT3 levels were measured using chemiluminescence at the Fujian provincial cancer hospital.

### Outcomes

The reference range of serum TSH, FT4, and FT3 were 0.34–5.6 μIU/mL, 9.1–19.24 pg/mL, and 4.34–7.2 pg/mL, respectively. The primary outcome in this study was subclinical hypothyroidism, defined as serum TSH > 5.6 μIU/mL and serum FT4 within a reference range [8]. Clinical hypothyroidism was defined as serum TSH > 5.6 μIU/mL and serum FT4 < 9.1 pg/mL, was the second outcome of the study.

### Statistical analysis

The non-parametric Kruskal–Wallis test and *t* test were used to compare non-normal and normal continuous variables between groups, respectively. The Chi-Square test was used for nominal variables. Survival data were analyzed using log-rank and cox regression analysis. All statistical analyses were conducted with SPSS 19.0 software. Sample size calculation was performed by PASS 15.0. *P* values (two-tailed) < 0.05 were considered to indicate statistical significance.

## Results

As shown in Table 1, there were no substantial differences in baseline or clinical characteristics between the two groups. The mean age of the thyroid dose-volume constraint group and the non-restricted group were 46.43 and 47.24 years old, respectively.

**Table 1:** Baseline and clinical characteristics of the thyroid dose-volume constraint versus the non-restricted groups

Variables	Thyroid dose-volume constraint		*P* value
	Yes (N = 46)	No (N = 46)	
Male [n (%)]	34 (73.9)	34 (73.9)	1.000
Age (years, mean ± SD)	46.43 ± 7.85	47.24 ± 9.65	0.662
T stage [n (%)]			0.652
T0	2 (4.3)	0 (0.0)	
T1	15 (32.6)	14 (30.4)	
T2	8 (17.4)	7 (15.2)	
T3	14 (30.4)	17 (37.0)	
T4	7 (15.2)	8 (17.4)	
N stage [n (%)]			0.705
N0	6 (13.0)	4 (8.7)	
N1	18 (39.1)	23 (50.0)	
N2	13 (28.3)	10 (21.7)	
N3	9 (19.6)	9 (19.6)	
FT3 on admission (pmol/L, mean ± SD)	5.45 ± 0.62	5.32 ± 0.58	0.315
FT4 on admission [pmol/L, M (IQR)]	11.46 (10.40–12.68)	11.04 (10.52–12.00)	0.870
TSH on admission [mIU/L, M (IQR)]	1.37 (0.54–4.30)	1.48 (1.12–3.13)	0.472
Thyroid volume before radiotherapy [cc, M (IQR)]	13.91 (11.29–17.58)	13.20 (11.26–16.23)	0.437
PTV coverage [%, M (IQR)]	98.4 (97.9–98.95)	98.6 (97.85–98.95)	0.841
Mean dose received by spinal cord (Gy, mean ± SD)	39.6 ± 1.28	40.38 ± 0.63	0.306
Mean dose received by esophagus (Gy, mean ± SD)	38.54 ± 3.11	41.48 ± 4.65	0.322
No	14 (30.4)	13 (28.3)	
Concurrent chemoradiotherapy [n (%)]			0.815
Yes	14 (32.6)	14 (30.4)	
No	29 (66.7)	29 (69.0)	
Adjuvant chemotherapy [n (%)]			0.335
Yes	4 (8.7)	7 (15.2)	
No	42 (91.3)	39 (84.8)	
Radiotherapy technology [n (%)]			0.404
VMAT	21 (45.7)	25 (54.3)	
TOMO	25 (54.3)	21 (45.7)	
Targeted therapy			0.877
Yes	25 (58.1)	26 (56.5)	
No	18 (41.9)	20 (43.5)	

At the time of last follow-up on August 31st, 2022, 9 patients in the thyroid dose-restricted group had subclinical hypothyroidism and 8 had clinical hypothyroidism. The number of subclinical hypothyroidism and clinical hypothyroidism was about twice as high in the non-dose restriction group as in the restriction group. Significant difference in the incidence of subclinical hypothyroidism between the thyroid dose-restricted group and non-restricted group was observed (*P* = 0.023). While statistical difference in the incidence of clinical hypothyroidism between the two groups was not seen (*P* = 0.058) (Table 2).

**Table 2:** Subclinical and clinical hypothyroidism occurred in both groups

Variable	Thyroid dose-volume constraint		*P* value
	Yes (N = 46)	No (N = 46)	
Subclinical hypothyroidism [n (%)]			0.023
Yes	9 (19.6)	19 (41.3)	
No	37 (80.4)	27 (58.7)	
Clinical hypothyroidism [n (%)]			0.058
Yes	8 (17.4)	16 (34.8%)	
No	38 (82.6)	30 (65.2)	

**Fig. 1:**
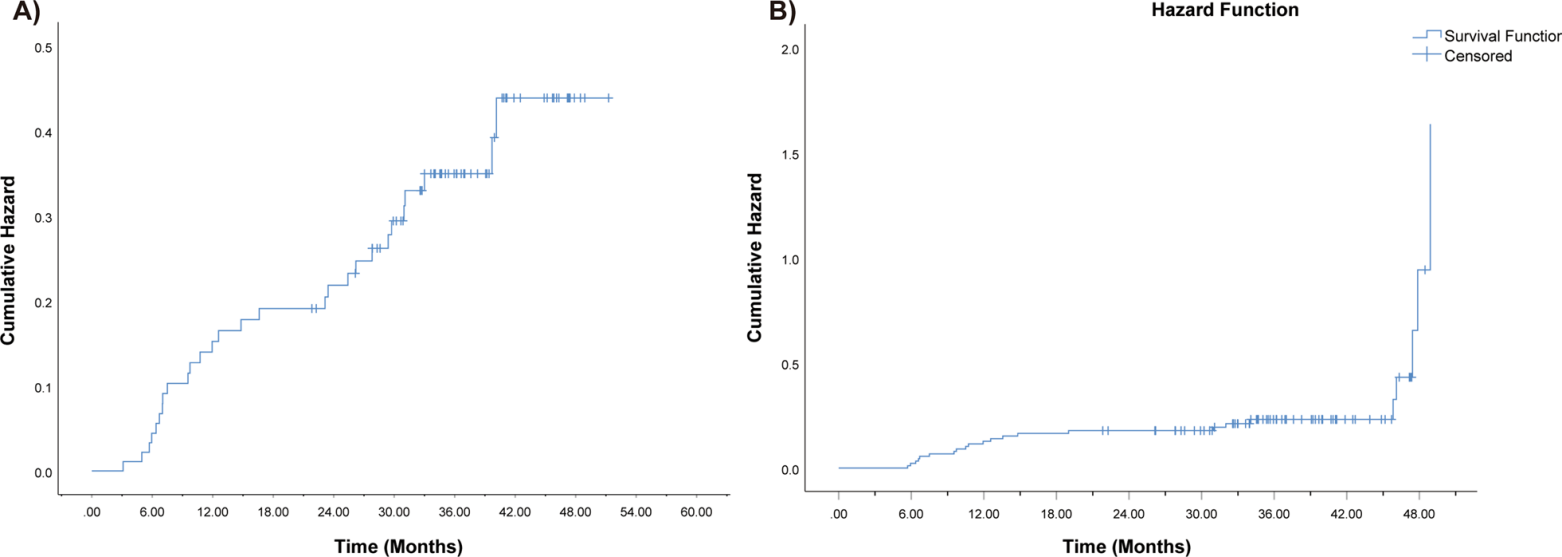
The cumulative incidence of subclinical (**a**) and clinical (**b**) hypothyroidism

As shown in Fig. 1a, the cumulative incidence of subclinical HT in patients was 20%, 31%, and 37% at 1, 2, and 3 years, respectively. And the cumulative incidence of clinical HT at the same time points was 11.2%, 16.3%, and 20.6%, respectively (Fig. 1b).

**Fig. 2:**
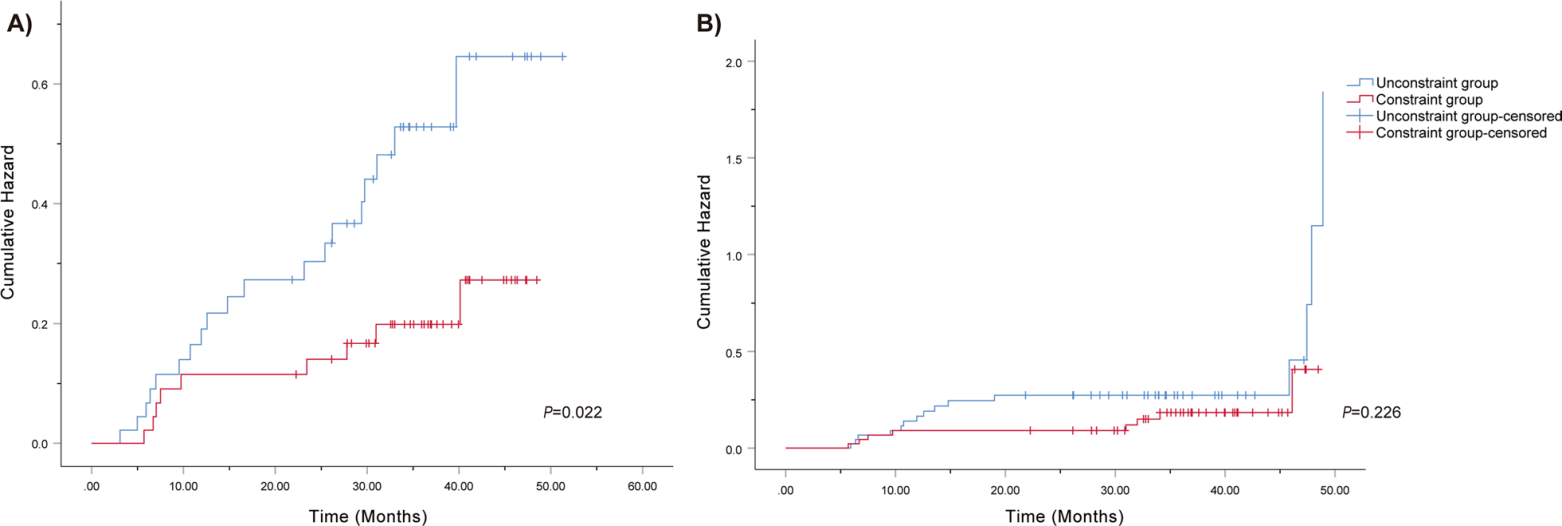
Kaplan–Meier curve for comparison of subclinical (**a**) and clinical (**b**) hypothyroidism hazard

As presented in Fig. 2a, the risk of subclinical hypothyroidism in the dose-limited group was lower than that in the unrestricted group (*P* = 0.022). The HR and HR_adjusted_ for subclinical hypothyroidism for thyroid dose-volume constraint versus non-dose-volume constraint were 0.408 (95% CI 0.184–0.904) and 0.361 (95% CI 0.155–0.841), respectively (Table 3). Similarly, the risk of clinical hypothyroidism was also higher in non-constraint group than in constraint group, but it was not statistically significant (*P* = 0.226) (Fig. 2b). After adjusting for sex, age, T stage, N stage, and radiotherapy technique, the risk of subclinical hypothyroidism in VS40 ≥ 3 cm group was lower than that in VS40 < 3 cm group (HR_adjusted_ = 0.714, 95% CI 0.574–0.887) (Table 3).

**Table 3:** Univariate and multivariate cox regression analysis of subclinical and clinical hypothyroidism

Variables	Subclinical hypothyroidism		Clinical hypothyroidism	
	Crude HR (95% CI)	Adjusted HR (95% CI)^a^	Crude HR (95% CI)	Adjusted HR (95% CI)^a^
Thyroid dose-volume constraint (yes vs. no)	0.408 (0.184–0.904)	0.361 (0.155–0.841)	0.585 (0.243–1.409)	0.537 (0.209–1.383)
VS40, per cm^3^ increase	0.834 (0.720–0.967)	0.714 (0.574–0.887)	0.972 (0.862–1.095)	0.888 (0.749–1.051)
VS40 (≥ 3 cc vs. < 3 cc)	0.442 (0.210–0.930)	0.295 (0.121–0.721)	0.670 (0.289–1.557)	0.380 (0.135–1.070)

VS40, the absolute volume of the thyroid spared from 40 Gy

^a^Adjusted for sex, age, T stage, N stage, and radiotherapy technology

## Discussion

With the widespread use of intensity-modulated radiation therapy (IMRT) in recent decades, most patients with nasopharyngeal carcinoma (NPC) are now able to survive long-term. However, NPC survivors still face a number of long-term sequelae that have many negative effects on their quality of life. One of the most common late toxicities is hypothyroidism (HT). It is reported to occur in 40–50% of patients treated with neck irradiation [6, 14]. Severe hypothyroidism can induce cardiac and cognitive dysfunction and depression. Therefore, exploring an effective dose constraint indicator for an IMRT planning is essential to improve the quality of life for NPC patients.

A single center randomized clinical trial was completed to determine whether V40 ≤ 85% can be used as an effective dose constraint in IMRT planning. The cumulative incidence of subclinical HT in patients was 20%, 31%, and 37% at 1, 2, and 3 years, respectively. A total of 28 patients (30.4%) developed subclinical HT, similar to previous study [15]. Nine of 46 people (19.6%) in the dose-volume constraint group developed subclinical hypothyroidism during follow-up, as compared with 19 (41.3%) in the unrestricted group. The risk of subclinical hypothyroidism in the dose-volume constraint group was lower than that in the unrestricted group (*P* = 0.022). Univariate and multivariate cox regression analysis indicated that thyroid dose-volume constraint was significantly associated with subclinical HT.

In our study, consistent with a previous study by Sommat et al. [13], patients in the dose-volume constraint (V40 ≤ 85%) group had a lower risk of subclinical HT than in the unrestricted group. This seems different in several studies. A recent study [16] suggested V40 < 80% to be an optimal dose-constraint index in their study of 404 non-metastatic NPC patients (AUC = 0.631). The rate of hypothyroidism in patients with mean thyroid dose < 45 Gy was significantly lower than that in patients with mean thyroid dose ≥ 45 Gy (31.9% vs 49.8%, *P* < 0.05). Therefore, they suggested Dmean < 45 Gy, V40 < 80% or VS45 ≥ 5 cm^3^ should be used as optimal limiting targets for IMRT treatment. Zhu et al. suggested V35 (3 Gy) < 58% to the thyroid gland can be used as the index of optimizing IMRT regimen to reduce the incidence of HT in patients with NPC [7]. Another study [17] presented that thyroid volume ≤ 20 cm^3^, thyroid V30,60 ≤ 80% might be effective dose constraints used in IMRT planning. Nevertheless, prospective randomized clinical trials are needed to determine whether all of the indexes can be used as optimal dose-constraint in IMRT planning.

Our study indicated the importance of regular follow-up as well as testing of thyroid function in NPC patients after radiotherapy. Usually, Patients with subclinical hypothyroidism occurs insidiously and lack specific symptoms. Symptoms in patients with subclinical hypothyroidism are mainly characterized by reduced metabolic rate and decreased sympathetic excitability, such as fatigue, memory loss, constipation, and slow pulse rate. Rarely, coma with mucous oedema occurs in patient with severe subclinical hypothyroidism. Furthermore, according to the guideline about subclinical hypothyroidism [18], L-T4 substitute therapy is recommended for patients with severe subclinical hypothyroidism (TSH ≥ 10 mIU/L). Patients with mild subclinical hypothyroidism (TSH < 10 mIU/L) should be treated with L-T4 if accompanied by symptoms of hypothyroidism, TPOAb positivity, dyslipidemia or atherosclerotic disease. According to National Comprehensive Cancer Network (NCCN) Guidelines, thyroid function tests are recommended every 6–12 months for patients with hypothyroidism in head and neck cancer.

The overall survival rate for patients with NPC is higher than for other head and neck tumors, which means that quality of survival associated with hypothyroidism draws the attention for NPC patients after radiotherapy. Therefore, it is important to explore whether thyroid V40 ≤ 85% can be used as an effective dose constraint in IMRT planning. However, the outcomes cannot be extrapolated to other entities where the neck receives bilateral irradiation, such as oral cavity tumors, oropharynx and hypopharynx cancers. As the planning target volume of other neck cancers usually include a portion of the VI lymph nodes, the actual irradiation dose to the thyroid gland in patients with other neck cancers is higher than NPC patients. Moreover, the dose of irradiation of the drainage area of the cervical lymph nodes in patients with other neck cancers, such as laryngeal cancer, is different from that of NPC patients. Therefore, to ensure the efficacy of treatment for other head and neck cancers, the thyroid should not be unduly restricted.

The main strength of our study is that it is the first randomized clinical trial that explored the HT induced by radiotherapy in NPC in a follow-up period of 34 months. However, limitations of the study also need to be considered. First, this is a single-center study with a small sample size. The recommended samples were taken as per sample size calculation to provide adequate power to detect significance. However, larger, multi-center randomized clinical trials are needed to generalize the result. Second, this study did not evaluate the quality of life of patients. Further study will be conducted in the future.

## Conclusion

This study indicated that V40 ≤ 85% can be used as an effective dose constraint in IMRT planning to prevent radiation-induced HT. This finding warrants further evaluation in a larger multicenter randomized clinical trial.

## Data Availability

Not applicable.
